# Exosome miRNA sorting controlled by RNA-binding protein-motif interactions

**DOI:** 10.20517/evcna.2025.47

**Published:** 2025-08-11

**Authors:** Zixuan Huang, Xuan Zhao, Wenjing Wen, Ruolin Shi, Gaofeng Liang

**Affiliations:** ^1^Henan International Joint Laboratory of Small Nucleic Acid and Tumor Precision Theranostics, School of Basic Medicine and Forensic Medicine, Henan University of Science and Technology, Luoyang 471023, Henan, China.; ^2^Henan Academy of Science, Zhengzhou 450000, Henan, China.

**Keywords:** Extracellular vesicles, RNA-binding proteins, EXOmotifs, CELLmotifs, microRNA sorting, engineered extracellular vesicles

## Abstract

Exosomes, as key mediators of intercellular communication, have shown great potential in disease intervention and therapy in recent years. As natural nanocarriers, exosomes play a crucial role in transporting a wide array of cargo. Among these, miRNAs carried by exosomes are pivotal in gene regulation and the modulation of cellular signaling. Given that miRNAs are essential gene regulators, understanding how miRNAs are selectively loaded into exosomes is crucial for the development of novel diagnostic and therapeutic approaches. This review provides a detailed overview of the biogenesis and secretion mechanisms of exosomes, with a particular focus on the molecular mechanisms governing miRNA sorting into exosomes. Specifically, it highlights the miRNA motifs associated with exosomes enrichment (EXOmotifs), as well as those related to intracellular miRNA enrichment (CELLmotifs), along with RNA-binding proteins (RBPs) involved in sorting. We summarize the current progress in this field and discuss strategies for engineering Exosomes - such as gene editing, drug loading, and surface modification - to enhance their functionality and specificity. By exploring these mechanisms, this review offers a theoretical foundation for the application of engineered exosomes in disease treatment and outlines future research directions and potential applications.

## INTRODUCTION

Exosomes are a specific subset of EVs (extracellular vesicles), characterized by their small size (30-150 nanometers in diameter) and a lipid bilayer membrane structure^[1]^. Exosomes are secreted by most cell types, including immune cells, epithelial cells, neurons and stem cells, and they are recognized as a critical component of intercellular communication^[2-4]^. They function in diverse biological processes and influence the behavior of recipient cells, making them valuable biomarkers and potential therapeutic agents. Detailed functional roles of exosomes in disease processes are further discussed in later sections. These exosomes transport a wide array of molecular cargo, such as proteins, lipids, mRNA, microRNA (miRNA), and DNA^[5-8]^. The biomolecules carried by exosomes can reflect the physiological state of the parent cell and influence the behavior of recipient cells, making them valuable biomarkers for disease diagnosis and prognosis^[9-12]^. For example, miRNAs present in exosomes have emerged as powerful diagnostic tools^[13,14]^. The presence of specific miRNAs in blood-derived exosomes can serve as early biomarkers for various cancers, providing a non-invasive means of detecting malignancies before they become clinically evident^[15-18]^. Typically, exosomes are involved in the transfer of these molecular signals to target cells through bodily fluids like blood, lymph, cerebrospinal fluid, and urine^[14,18]^. Once taken up by recipient cells, the miRNAs and other molecules influence the cellular machinery, regulating gene expression, protein synthesis, and cellular responses, thereby modulating the physiological state and functionality of the target cells^[19,20]^. The ability of exosomes to mediate intercellular communication and influence recipient cellular behavior is of particular interest for the development of new therapeutic strategies^[21]^.

Understanding the molecular mechanisms underlying the loading of specific miRNAs and other biomolecules into exosomes is critical for advancing their clinical applications. In particular, the role of RNA-binding proteins (RBPs) in the sorting and packaging of miRNAs into exosomes has become a key area of investigation^[22]^. These RBPs interact with miRNAs and facilitate their incorporation into exosomes, ensuring that only specific miRNAs are selected for packaging and transport. The recognition motifs within miRNAs, known as EXOmotifs (associated with exosomes enrichment) and CELLmotifs (related to intracellular enrichment), play a pivotal role in this sorting process^[23]^. Moreover, RBPs that recognize these motifs are essential for the selective loading of miRNAs into exosomes^[22]^. This review will delve into the current research on the interactions between RBPs, miRNAs, and exosomes, providing a comprehensive understanding of the molecular mechanisms involved in miRNA sorting and packaging. Additionally, we will explore the potential of engineered exosomes, which can be modified through gene editing, drug loading, and surface modification techniques, to enhance their therapeutic capabilities and target specificity. By manipulating these mechanisms, engineered exosomes could be used as a novel class of therapeutic vehicles for the targeted delivery of drugs, nucleic acids, and other bioactive molecules to diseased tissues. Furthermore, we will examine how the RBP-miRNA-exosomes axis can be leveraged for the development of new diagnostic and therapeutic approaches, with a particular focus on disease treatment strategies that aim to exploit the natural properties of exosomes.

This review also aims to provide insights into the future directions of exosome-based research, including the challenges and opportunities associated with harnessing exosomes for clinical applications. As our understanding of the complex biology of exosomes continues to evolve, these vesicles hold the promise of transforming personalized medicine, offering novel ways to diagnose, monitor, and treat a wide range of diseases.

## FORMATION AND REGULATORY DYNAMICS OF EXOSOMES

### Molecular mechanisms underlying exosome formation

**Figure 1:**
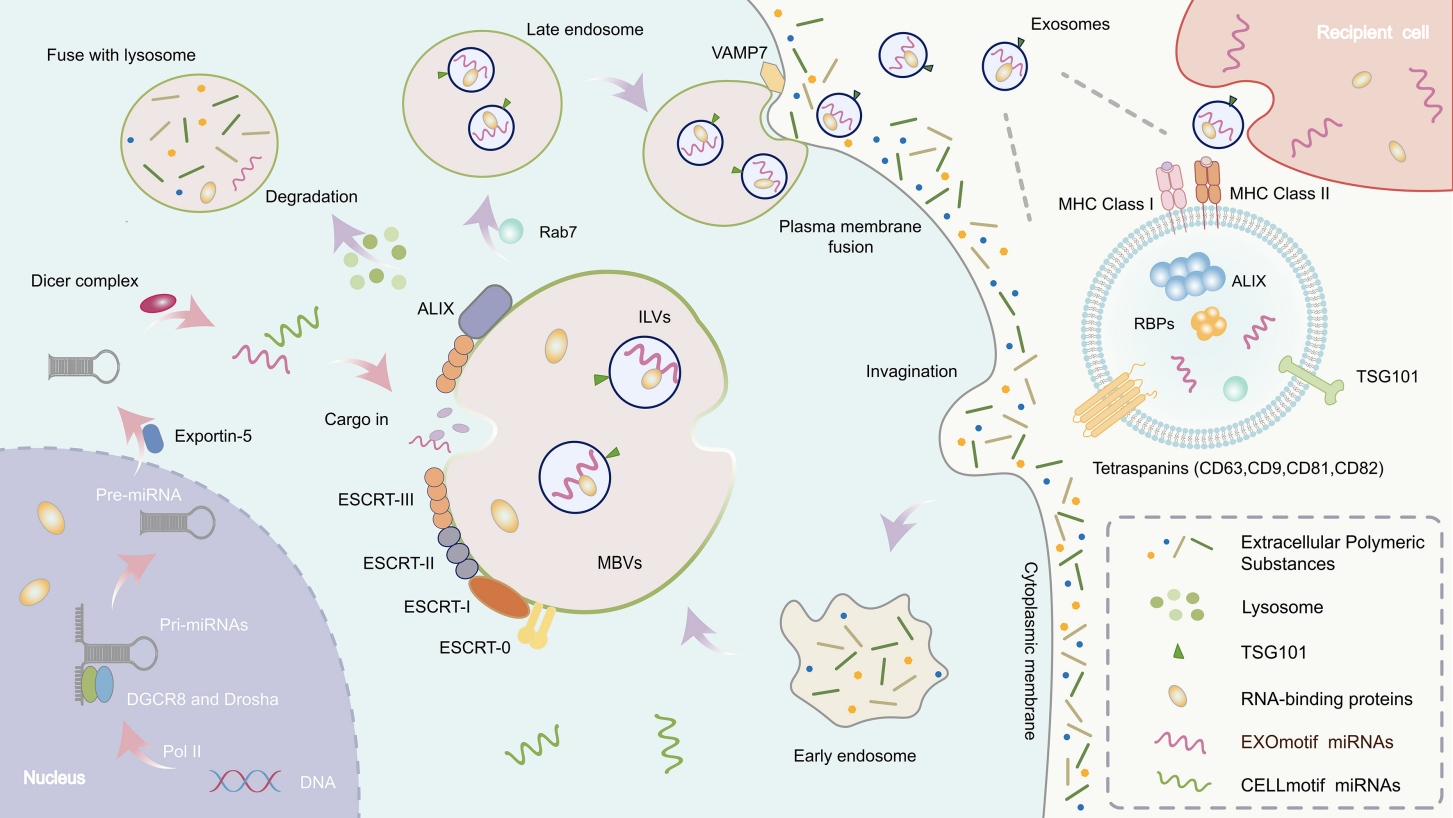
Exosome formation and miRNA biogenesis. The left section depicts the biogenesis of miRNAs. In the nucleus, pri-miRNAs are transcribed by RNA polymerase II and processed by the Microprocessor complex (Drosha and DGCR8) into pre-miRNAs. These pre-miRNAs are exported to the cytoplasm via Exportin-5 and further processed by Dicer into mature miRNAs. Specific RBPs can recognize sequence motifs on miRNAs (e.g., EXOmotifs) and mediate their selective loading into MVBs. miRNA: MicroRNA; pri-miRNA: primary microRNA; pre-miRNA: precursor microRNA; RBP: RNA-binding protein; MVB: multivesicular body; RNA Pol II: RNA polymerase II; DGCR8: DiGeorge syndrome critical region gene 8; ESCRT: endosomal sorting complex required for transport; ILV: intraluminal vesicle; VAMP7: vesicle-associated membrane protein 7; MHC: major histocompatibility complex.

Exosome biogenesis and secretion represent an intricate, highly regulated process that involves a multitude of organelles, signaling pathways, and molecular mechanisms. This process begins with the endocytic pathway, wherein the cell membrane engulfs extracellular material, including lipids, proteins, and other signaling molecules, to form early endosomes^[24]^. These early endosomes are dynamic structures that serve as the precursor to more specialized endosomal compartments, progressing into late endosomes. Late endosomes, commonly referred to as multivesicular bodies (MVBs), are key intermediates in the formation of exosomes [Figure 1].

The transformation from early to late endosomes is marked by a series of molecular changes, including the conversion of Rab5, a small GTPase, to Rab7, which serves as a crucial marker for endosome maturation^[25]^. As late endosomes mature, their internal membranes undergo a process of inward budding to form intraluminal vesicles (ILVs)^[26,27]^ [Figure 1]. This membrane invagination is essential for the eventual formation of exosomes^[26]^. The process of ILV formation is tightly regulated by the ESCRT (endosomal sorting complex required for transport) machinery, which plays a critical role in sorting specific proteins, lipids, and RNA into the developing vesicles^[28]^. The ESCRT machinery is composed of several protein complexes, namely ESCRT-0, ESCRT-I, ESCRT-II, and ESCRT-III, each performing distinct functions in the sorting, budding, and scission of vesicles^[29,30]^. Notably, ESCRT-III is particularly important for the membrane scission event, where the inner membrane of the endosome is pinched off to release ILVs^[30,31]^ [Figure 1]. Meanwhile, ESCRT-0, ESCRT-I, and ESCRT-II are primarily involved in cargo sorting and the recruitment of ubiquitinated proteins to nascent vesicles^[30,32]^. Interestingly, some studies suggest that MVB formation may occur through ESCRT-independent pathways, mediated by the cytoskeleton and lipid rafts, which are specialized membrane microdomains enriched with cholesterol and sphingolipids^[33]^. These alternative pathways may contribute to the formation of exosomes in specific cellular contexts and have been implicated in certain pathological conditions, such as cancer and neurodegenerative diseases^[34]^. The dynamic interplay between ESCRT-dependent and ESCRT-independent mechanisms highlights the complexity of the exosome biogenesis process^[33]^.

Once MVBs are formed, they are directed toward two potential fates: they can either fuse with lysosomes for degradation or, under specific signaling conditions, fuse with the plasma membrane, releasing their contents into the extracellular space. This release is the final step in exosome secretion [Figure 1]. The process by which MVBs are transported to the cell membrane and subsequently fuse with it is complex and involves several regulatory factors, including motor proteins, small GTPases, and various molecular interactions^[24,27]^. The movement of MVBs toward the plasma membrane is primarily mediated by cytoskeletal motor proteins, which utilize the microtubule network as tracks for movement^[27,35]^. Small GTPases, particularly members of the Rab family, such as Rab27a and Rab27b, play a pivotal role in regulating the movement, docking, and localization of MVBs to the cell membrane^[36]^. These proteins control the trafficking of MVBs within the cell, ensuring that the vesicles are accurately directed to the plasma membrane for fusion. The fusion of MVBs with the plasma membrane is a critical step in exosome secretion, and it requires the coordination of a variety of proteins. One of the most essential groups of proteins involved in membrane fusion are the SNARE (soluble N-ethylmaleimide-sensitive factor attachment protein REceptor) proteins, such as VAMP7 (vesicle-associated membrane protein 7)^[37,38]^. SNARE proteins facilitate the merging of the MVB membrane with the cell membrane, allowing for the release of ILVs into the extracellular space^[38]^ [Figure 1]. The release of ILVs is what ultimately results in the formation of exosomes, which are small vesicles that carry a cargo of proteins, lipids, and RNA molecules, and can participate in intercellular communication.

The Middle (MID) section shows the formation of exosomes, which begins with the inward budding of the plasma membrane to form early endosomes. These mature into late endosomes or MVBs. The membrane of MVBs invaginates to generate ILVs. Upon fusion of MVBs with the plasma membrane, ILVs are released as exosomes, while some MVBs are directed to lysosomes for degradation.

The right section is a magnified view of the exosome structure. This process is regulated by the ESCRT complex (ESCRT-0, -I, -II, -III) and tetraspanins (such as CD9, CD63, CD81, and CD82).

This figure highlights the coupling between exosomal trafficking and miRNA processing mechanisms, emphasizing the crucial role of RBPs in selectively packaging miRNAs into exosomes, thereby facilitating intercellular communication.

### Key players in exosome biogenesis

Regulation of exosome secretion is influenced by a range of factors, including calcium ions, cellular stress, and metabolic states^[39]^. Calcium ions are particularly important in this process, as they can activate calcium-dependent proteins, such as synaptotagmin, which promote the fusion of MVBs with the plasma membrane. Calcium signaling also regulates various aspects of vesicle trafficking, including vesicle docking and fusion events. Further, cellular stressors, such as hypoxia, oxidative stress, or heat shock, can induce changes in the endocytic and exocytic pathways, leading to increased exosome secretion^[40-43]^. Under stress conditions, cells may alter their membrane fusion mechanisms to release more exosomes, which could help in modulating the cellular environment or facilitating stress response signaling^[40]^. Metabolic states also play a significant role in regulating exosome production. Alterations in cellular metabolism, such as those occurring in cancer cells, can lead to changes in the quantity and cargo composition of exosomes^[44]^. In cancer, exosomes can carry oncogenic factors that promote tumor growth and metastasis, highlighting the importance of exosomes-mediated signaling in disease progression^[45]^.

To sum up, exosome biogenesis and secretion are a highly orchestrated process that involves a multitude of molecular players and cellular machinery. From the formation of early endosomes to the maturation of MVBs and their subsequent fusion with the plasma membrane, each step is meticulously regulated by various proteins, small GTPases, and lipid dynamics. The secretion of exosomes, which carry a diverse array of bioactive molecules, plays an essential role in intercellular communication and can be influenced by cellular stress, calcium signaling, and metabolic changes. The complexity and regulation of exosome secretion underscore their critical function in health and disease, and further understanding of these processes could have profound implications for therapeutic strategies targeting exosomes-mediated pathways.

## THE MULTIFACETED BIOLOGICAL ROLES OF EXOSOMES

### The multifaceted roles of exosomes in intercellular communication and disease regulation

Exosomes, due to their unique lipid bilayer structure, have emerged as key mediators of intercellular communication, playing essential roles in the transfer of bioactive molecules between cells. They transport diverse molecular cargos - including proteins, nucleic acids (notably miRNAs), lipids, and signaling molecules - that mediate intercellular communication^[46]^. Through mechanisms such as membrane fusion or receptor-mediated endocytosis, exosomes deliver their cargos to recipient cells, thereby influencing gene expression, signal transduction, and a wide range of physiological and pathological processes. The bioactive molecules carried by exosomes are involved in regulating gene expression, cellular signaling, and several critical processes such as proliferation, differentiation, migration, apoptosis, and immune responses.

Among the various bioactive components, miRNAs - small non-coding RNAs central to post-transcriptional gene regulation - represent a major mechanism by which exosomes influence target cell behavior^[47]^. Through the selective shuttling of specific miRNAs, exosomes can alter gene expression patterns in recipient cells. For instance, tumor-derived exosomes carry oncogenic miRNAs that are taken up by immune cells within the tumor microenvironment. These miRNAs suppress immune responses and facilitate immune evasion, thereby contributing to tumor progression and metastasis^[47]^. In contrast, cardiomyocyte-derived exosomes can deliver protective miRNAs that facilitate myocardial repair, underscoring their therapeutic potential in cardiovascular diseases^[48]^. In the context of neurodegenerative diseases, exosomes have been identified as carriers of pathological proteins such as tau and amyloid-beta, which are associated with Alzheimer’s disease, making them promising biomarkers for disease monitoring and progression^[34]^.

Beyond their involvement in cancer and tissue repair, exosomes act as pivotal mediators of immune regulation, inflammation, and cellular stress responses^[49]^. Exosomes secreted by immune cells can transport antigen-presenting molecules and cytokines, thereby playing a central role in modulating both innate and adaptive immune responses. These immune-related exosomes contribute to T cell activation, antigen presentation, and regulation of inflammation^[50]^. Moreover, under stress conditions such as oxidative stress or hypoxia, cells can release exosomes containing stress-associated factors that propagate adaptive responses to neighboring cells, promoting tissue-wide coordination.

Altogether, exosomes are increasingly recognized for their multifaceted functions in physiological regulation and disease progression, and their growing association with various diseases has spurred the development of emerging therapeutic strategies. These advances highlight the potential of exosomes not only as carriers of diagnostic biomarkers but also as promising vehicles for targeted drug delivery, offering new avenues for treating complex diseases.

### Therapeutic potential of exosomes in drug delivery

Exosomes show significant promise in therapeutic applications, especially as drug delivery systems. Their natural ability to encapsulate and protect bioactive molecules makes them ideal candidates for targeted delivery. However, despite many studies highlighting the potential of exosomes in drug delivery, the understanding of the precise mechanisms that govern the selective sorting of therapeutic molecules into exosomes is still limited. This is particularly true when it comes to understanding how RNA and protein cargo are loaded into exosomes and how these molecules manage to escape endosomal encapsulation in recipient cells. Given these gaps in knowledge, one might question whether the current enthusiasm for exosomes’ potential could be overstating their therapeutic efficacy, especially considering the technical challenges in fully understanding cargo sorting mechanisms and the variable production of exosomes under different conditions.

Recent studies have provided valuable insights into the role of exosome-derived miRNAs in disease progression, shedding light on their diagnostic and therapeutic potential. For instance, research has shown that exosomes released from damaged podocytes can induce apoptosis and p38 phosphorylation in renal tubular epithelial cells, a process mediated by specific miRNAs such as miRNA-424 and miRNA-149^[51,52]^ [Table 1]. These miRNAs play a crucial role in renal tubular damage, which is commonly observed in glomerular diseases. This finding highlights how exosomal miRNAs can influence disease progression, particularly in kidney-related pathologies. While this example underscores the potential of exosomes in diagnostics and targeted therapy, it also emphasizes the need to better understand how miRNA cargoes are sorted, packaged, and delivered within exosomes, which remains a critical gap in exosome-based therapeutic strategies^[53]^.

**Table 1:** Disease-related functions of miRNAs encapsulated in extracellular vesicles

**MiRNA cargo**	**Target cell/tissue**	**Associated disease(s)**	**Functional role/mechanism**	**References**
miR-483-5p	Renal tubular epithelial cells (HK2)	Renal interstitial fibrosis	Delays fibrosis progression	[102]
miR-27b-3p	Endothelial cells	Increased vascular permeability	Enhances vascular permeability	[103]
miR-196a	Cancer-associated cells	Head and neck cancer	UAGGUA motif promotes selective loading	[104]
miR-320	Bone marrow niche	Leukemia	Reshapes bone marrow microenvironment	[105]
miR-522	Tumor cells	Tumor progression	Suppresses ferroptosis, promotes tumor growth	[106]
miR-1246	Neural cells	Glioblastoma	Associated with postoperative recurrence	[107]
miR-424, miR-149	HK2 cells	Glomerular disease	Induces apoptosis and p38 phosphorylation	[51,52]
miR-208a, miR-208b	Cardiomyocytes	Cardiac injury	Targets QKI, promotes apoptosis	[135]
miR-146a	Immune cells	Inflammatory regulation	Negatively regulates NF-κB signaling by targeting *TRAF* and *IRAK* genes, suppressing pro-inflammatory cytokine activation	[75]
miR-1246	Circulating system	Gastric cancer	HuR-mediated enrichment, linked to metastasis	[117,118]
miR-125b	Neurons	Neurodegenerative (potential)	FMR1 recognizes hairpin structure	[140]
miR-155	Inflammatory cells	Inflammatory diseases	Regulates inflammatory responses via the AAUGC motif and the ESCRT pathway	[141]

miRNA: MicroRNA; HK2: human kidney 2; UAGGUA: uracil-adenine-guanine-guanine-uracil-adenine; QKI: quaking protein; NF-κB: nuclear factor kappa-light-chain-enhancer of activated B cells; TRAF: TNF receptor-associated factor; IRAK: interleukin-1 receptor-associated kinase; HuR: human antigen R; FMR1: fragile X mental retardation 1 protein; AAUGC: adenine-adenine-uracil-guanine-cytosine; ESCRT: endosomal sorting complexes required for transport.

By modifying their surface properties, exosomes can be engineered to deliver therapeutic molecules, such as small-molecule drugs, proteins, or nucleic acids, to specific cells or tissues^[54]^. For instance, exosomes can be designed to target specific receptors on diseased cells, ensuring precise delivery of the therapeutic cargo to the right location, while minimizing off-target effects^[55]^. This targeting capability enhances the efficacy of drugs while reducing potential side effects. However, due to the broad targeting ability of some surface-modified exosomes, further research is necessary to refine these targeting mechanisms and ensure that specificity is maintained during the engineering process.

One of the key advantages of using exosomes for drug delivery is their ability to cross biological barriers, such as the blood-brain barrier, which often limits the effectiveness of traditional drug delivery methods. Engineered exosomes can be designed to carry RNA-based therapies, such as siRNA or mRNA, that regulate gene expression within target cells^[55]^. This opens up new possibilities for treating genetic disorders, cancers, and other diseases that involve dysregulated gene expression. However, challenges remain in achieving efficient and stable RNA delivery into target cells, as the uptake and internalization efficiency of RNA-loaded exosomes are still suboptimal. Further research is needed to improve the stability of RNA within exosomes and the mechanisms by which RNA escapes from exosomes once inside cells. Additionally, exosomes can be loaded with small-molecule drugs or proteins that promote tissue regeneration, such as growth factors or anti-inflammatory cytokines, providing a potential therapeutic strategy for tissue repair following injury or in degenerative diseases. Techniques like gene editing, surface modification, and lipid-based formulations enable researchers to engineer exosomes with specific targeting capabilities and enhanced stability, making them even more effective as drug delivery vehicles^[56]^. For example, incorporating surface proteins or ligands into the EV membrane can direct the vesicles to specific cell types or tissues, such as tumor cells or immune cells^[57]^. Furthermore, EV-based drug delivery systems can be designed to release their cargo in response to specific stimuli, such as pH or temperature changes, providing controlled release of therapeutic agents. However, the precise mechanisms governing the release of EV cargo in response to these stimuli remain poorly understood, and additional research is needed to refine these mechanisms to make exosomes more predictable in their behavior. Beyond drug delivery, engineered exosomes are also being explored in gene therapy. By loading exosomes with therapeutic nucleic acids, such as CRISPR-Cas9 constructs or gene-editing RNA, genetic material can be delivered directly to target cells to correct genetic mutations or regulate gene expression^[58,59]^. This approach holds particular promise for treating genetic disorders caused by single-gene mutations or for editing immune cells to enhance their ability to fight cancer. Yet, given the challenges associated with the specificity of miRNA or RNA delivery into exosomes, future research must focus on optimizing the loading capacity of engineered exosomes and overcoming issues such as off-target effects and cargo instability.

Exosomes are far more than simple vesicles. They are complex, dynamic carriers of molecular information that play vital roles in cellular communication, disease progression, and therapeutic interventions. Their ability to shuttle a diverse range of bioactive molecules makes them invaluable for diagnostic and therapeutic applications. However, the full potential of engineered exosomes is still being explored, and more research is needed to understand their limitations, particularly in clinical settings.

## THE MECHANISMS OF MIRNA BIOGENESIS AND THEIR IMPLICATIONS IN DISEASE PATHOGENESIS

### Molecular pathways of miRNA biogenesis

MiRNAs are small, non-coding RNAs of approximately 22 nucleotides in length that exert post-transcriptional control over gene expression by binding to the 3’ untranslated regions (UTRs) of target mRNAs, leading to translational repression or mRNA degradation^[60,61]^. This process is critical for fine-tuning cellular functions, as miRNAs are involved in regulating a wide variety of biological processes, including cell growth, differentiation, development, apoptosis, and immune responses. miRNAs exert their regulatory functions by either promoting the degradation of mRNA or inhibiting its translation, thereby modulating the levels of proteins in the cell. Through these mechanisms, miRNAs play central roles in maintaining cellular homeostasis and tissue integrity, influencing numerous physiological processes.

The biogenesis of miRNAs is tightly regulated and involves multiple steps, beginning in the nucleus and extending into the cytoplasm^[62]^. This process begins with RNA polymerase II transcribing miRNA genes, producing primary miRNAs (pri-miRNAs)^[63]^ [Figure 1]. These pri-miRNAs are long RNA transcripts, often containing hundreds to thousands of nucleotides, and are characterized by one or more hairpin-like structures. The next step involves the processing of the pri-miRNA into a shorter precursor miRNA (pre-miRNA), which is approximately 70 nucleotides long and still retains its hairpin structure. This processing is carried out by the Microprocessor complex, composed of two key components: Drosha (drosha ribonuclease III), an RNase III enzyme, and DGCR8 (DiGeorge syndrome critical region 8), a cofactor that aids in recognizing the primary transcript^[62]^. The pre-miRNA is then transported from the nucleus to the cytoplasm via the Exportin-5 protein, which utilizes the nuclear pore complex for this transfer^[64]^ [Figure 1].

Once in the cytoplasm, the pre-miRNA is further processed by the Dicer complex, an RNase III enzyme that cleaves the hairpin structure, producing a double-stranded RNA duplex. This results in a double-stranded RNA duplex, typically 21-23 nucleotides long, which is then unwound into single-stranded mature miRNA. The miRNA duplex is then unwound, and the single-stranded RNA fragment, known as the mature miRNA, is incorporated into the RNA-induced silencing complex (RISC)^[65]^. This complex is primarily composed of Argonaute (AGO) proteins, along with other auxiliary factors that facilitate miRNA-mediated gene silencing. The Ago protein binds to both the 5’ and 3’ ends of the miRNA, stabilizing the interaction and allowing it to guide the RISC to its target mRNA. Once the miRNA is paired with its target mRNA, the RISC can either cause the degradation of the mRNA or inhibit its translation, thereby downregulating the expression of specific genes. Beyond the basic biogenesis pathway, miRNA maturation is regulated by several factors, including post-transcriptional modifications such as N6-methyladenosine (m^6^A)^[66]^. The m6A-modified pri-miRNAs can be recognized and bound by specific RBPs, which influence various stages of miRNA processing^[67]^. HnRNPA2B1 (heterogeneous nuclear ribonucleoprotein A2/B1), Lin28 (Lin-28 homolog A), and SMAD family proteins interact with pri-miRNAs, modulating their processing efficiency and subsequent conversion into pre-miRNAs^[68,69]^.

HnRNPA2B1, in particular, has been shown to recognize m6A marks on pri-miRNAs and promote their processing, thus facilitating the maturation of miRNAs^[67,70]^. In addition, other modifications such as phosphorylation and methylation can regulate the activity of enzymes like Drosha, Dicer, and Ago proteins, further modulating miRNA biogenesis and function. QKI (quaking homolog, KH domain RNA binding), a member of the STAR (signal transduction and activation of RNA) family, is also involved in miRNA regulation^[71,72]^. Specifically, QKI-5 promotes the maturation of pri-miR-124-1 by binding directly to its KH domain, which facilitates its interaction with DGCR8 and enhances processing efficiency^[73]^. This interaction plays a crucial role in the processing of pri-miR-124-1 into its mature form.

While the fundamental steps of miRNA biogenesis are well established, how this process is differentially regulated across diverse cell types and how it is altered in various physiological and pathological contexts remain areas of active investigation.

### Impact of miRNAs on disease progression

MiRNAs are not only vital for regulating gene expression but also play critical roles in immune system regulation^[74]^. They influence the development, differentiation, and functional activity of a variety of immune cells, thereby impacting both innate and adaptive immune responses. For instance, miR-146a is known to negatively regulate the NF-κB (nuclear factor kappa-light-chain-enhancer of activated B cells) signaling pathway by targeting *TRAF* and *IRAK* genes, which are key activators of pro-inflammatory cytokine expression. By modulating NF-κB signaling, miR-146a serves as a feedback inhibitor that limits excessive inflammation and maintains immune homeostasis^[75]^ [Table 1]. Complementarily, miR-155 modulates immune cell differentiation and inflammatory responses, yet its role is complex and context-dependent - promoting inflammation in acute immune activation while also contributing to immune resolution in chronic inflammation^[76]^. However, it is important to note that the immune-regulatory effects of miRNAs can vary significantly across different cell types and pathological conditions. Furthermore, because each miRNA can potentially target multiple mRNAs, their functional specificity remains an active area of investigation, and interpretations should be made with caution.

In addition to immune regulation, miRNAs are involved in the development and progression of various diseases, such as cancer, cardiovascular disorders, neurodegenerative diseases, and metabolic syndromes. For example, miR-21 is frequently overexpressed in various cancers and is implicated in promoting tumor growth and metastasis by targeting tumor suppressor genes such as PTEN (phosphatase and tensin homolog deleted on chromosome 10) and TIMP3 (tissue inhibitor of metalloproteinases-3)^[77,78]^. However, the functional impact of miR-21 can vary across tumor types and may depend on the tumor microenvironment. Conversely, miR-126 plays a protective role in the cardiovascular system by promoting angiogenesis and endothelial cell survival, thereby improving vascular integrity and reducing atherosclerotic risk^[79]^. In neurodegenerative diseases, members of the miR-29 family have been shown to downregulate DNA methyltransferases, thereby altering epigenetic profiles and potentially supporting neuronal survival^[80]^. Nonetheless, their roles may be context-dependent and require further investigation.

Exosomes serve as critical vehicles for intercellular communication by enabling the transfer of miRNAs between cells. The sorting of miRNAs into exosomes is believed to be a regulated process involving specific molecular determinants, including RBPs, post-transcriptional modifications, and potentially disease-related signaling pathways. While several models of selective loading have been proposed, the mechanisms remain incompletely understood. A better understanding of these processes is essential for deciphering how EV-associated miRNAs contribute to physiological regulation and disease progression. MiRNAs are versatile regulators of gene expression involved in diverse biological processes. Their association with exosomes and involvement in immunity, disease modulation, and epigenetic regulation underscore their significance in maintaining homeostasis. However, translating these findings into clinical applications will require overcoming challenges related to specificity, delivery, and functional validation.

### Molecular determinants of miRNA loading into exosomes

**Figure 2:**
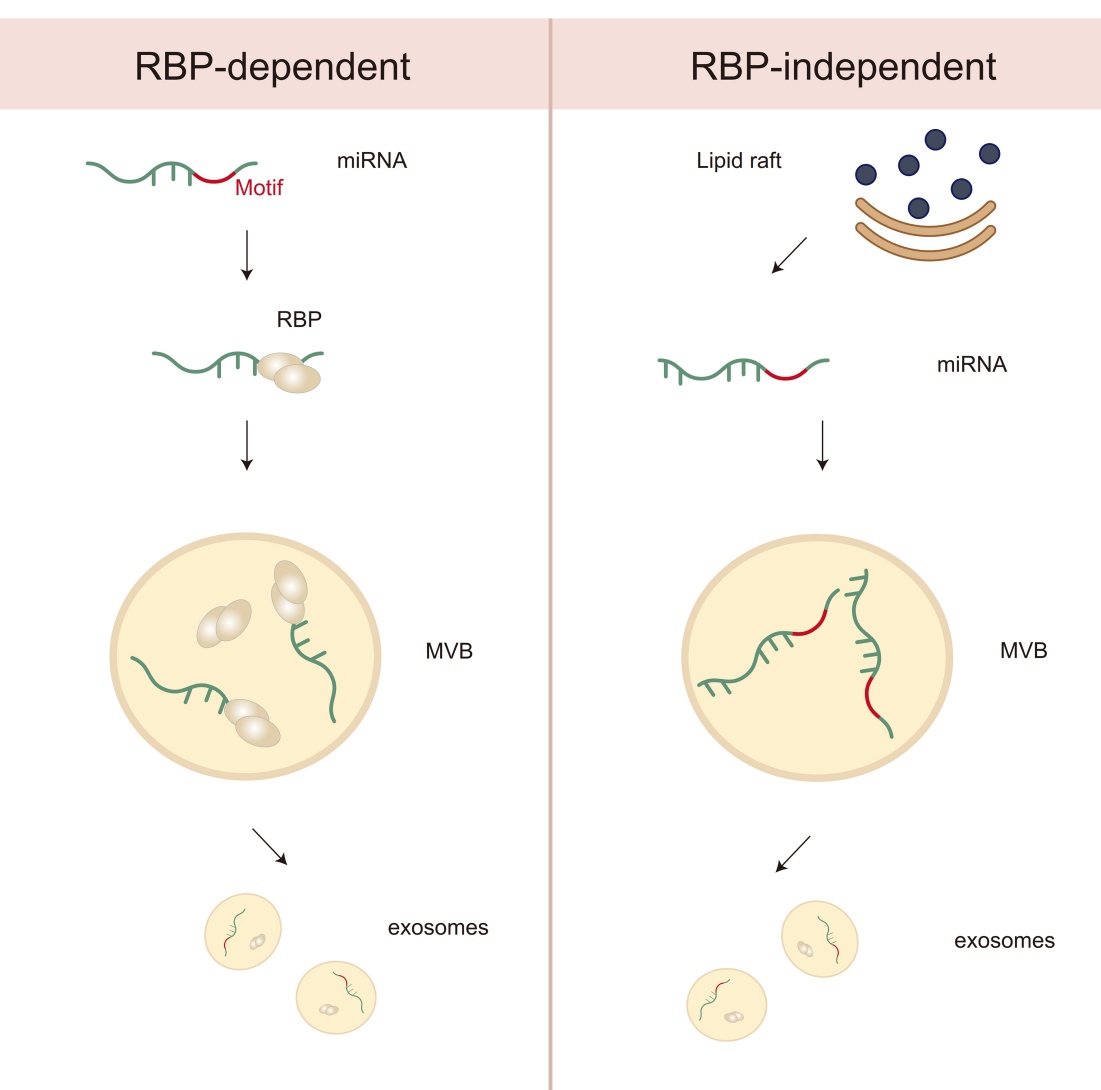
Differential mechanisms of miRNA sorting into exosomes: RBP-Dependent vs. RBP-Independent Pathways. This diagram illustrates two distinct mechanisms of miRNA sorting into exosomes. On the left, the RBP-dependent pathway involves the recognition of specific miRNA motifs by RBPs, which guide miRNAs to MVBs for incorporation into exosomes. On the right, the RBP-independent pathway is associated with lipid rafts, where miRNAs are incorporated into exosomes via membrane lipid interactions, bypassing the need for RBPs. Both pathways contribute to the selective packaging of miRNAs into exosomes for intercellular communication. miRNA: MicroRNA; RBP: RNA-binding protein; MVB: multivesicular body.

To fully harness the diagnostic and therapeutic potential of EV-miRNAs, it is essential to understand the underlying mechanisms by which these small RNAs are selectively sorted into vesicles. This section explores the molecular pathways governing miRNA loading into exosomes. The molecular mechanisms governing the sorting of miRNAs into exosomes are intricate and involve a range of cellular factors. These pathways can be broadly categorized into RBP-dependent and RBP-independent mechanisms [Figure 2]. At the core of RBP-dependent sorting are RBPs and specific miRNA sequence motifs, which together determine miRNA loading specificity and efficiency^[23]^. These RBPs not only bind to miRNA motifs but also interface with vesicle biogenesis machinery, thereby acting as adaptors linking miRNAs to the EV pathway. Examples include hnRNPA2B1, YBX1, and Ago2, each recognizing distinct motifs or modifications such as GGAG, UCAGU, or 5’/3’ end features, respectively. However, not all RBPs use the same recognition domains, and the relevance of sequence versus structural cues remains an area of active investigation.

In contrast, RBP-independent sorting mechanisms have been associated with lipid-based microdomains such as lipid rafts - cholesterol- and sphingolipid-enriched membrane regions that serve as platforms for the passive clustering or electrostatic capture of miRNAs^[81]^ [Figure 2]. Certain lipid species, such as phosphatidylserine (PS), have been shown to facilitate this process, although direct evidence for sequence-specific lipid–miRNA interactions remains limited. These non-protein-based pathways are less well understood and may vary depending on the cellular lipid composition and physiological context. The endosomal pathway, particularly involving MVB formation and inward budding of ILVs, is central to both sorting mechanisms [Figure 1]. The ESCRT machinery plays a major role in cargo selection and vesicle budding. ESCRT-0 subunits recognize ubiquitylated proteins and may indirectly sort RBPs associated with miRNAs, while ESCRT-III drives membrane deformation and scission to form ILVs^[81]^ [Figure 1]. Accessory proteins such as TSG101 and Alix further assist in coordinating the interaction between RBPs, membrane curvature, and cargo enrichment^[82]^ [Figure 1]. However, the precise mechanisms by which miRNAs and their binding partners are selectively included in ILVs remain incompletely defined and are likely influenced by multiple factors, including cellular stress, signaling pathways (e.g., KRAS, hypoxia), and RNA modifications (e.g., m^6^A).

Despite the progress made, several fundamental questions remain unresolved. For instance, it is still debated whether miRNAs can be directly sorted into exosomes independent of RBPs, or whether all sorting ultimately relies on intermediary protein adaptors. Moreover, current knowledge is largely derived from in vitro studies using bulk EV isolation methods, which may overlook heterogeneity at the single-vesicle level. Emerging technologies such as single-EV sequencing, crosslinking immunoprecipitation (CLIP), and lipidomics-based EV profiling may provide more mechanistic insight in the future.

In summary, miRNA sorting into exosomes is a highly coordinated and context-dependent process involving a complex interplay of RBPs, lipid microdomains, endosomal trafficking, and cellular state. A more complete understanding of these mechanisms is critical for improving EV engineering strategies, particularly for therapeutic applications requiring selective and reproducible miRNA loading. At the same time, overcoming technical barriers related to efficiency, scalability, and specificity remains essential for the clinical translation of EV-based miRNA delivery systems.

## RBPS AND THE SPECIFICITY OF MIRNA SORTING INTO EXOSOMES

Numerous studies have highlighted the significant role of miRNAs not only in regulating intracellular gene expression but also in their selective packaging into exosomes, thereby facilitating intercellular communication and signaling. The sorting of miRNAs into exosomes is a tightly regulated process that involves specific nucleotide motifs, known as miRNA motifs, which serve as signals for loading miRNAs into exosomes. These motifs are recognized by intracellular sorting mechanisms, involving specialized RBPs, which determine whether miRNAs are retained within the cell or transported in exosomes to other cells^[23]^.

One of the key factors in the selective sorting of miRNAs into exosomes is the presence of specific motifs such as EXOmotifs and CELLmotifs^[23]^. EXOmotifs are specific nucleotide sequences or structural elements found in miRNAs that are recognized by RBPs, facilitating their packaging into exosomes. For instance, two RBPs, Alyref and Fus, play a role in the export of miRNAs that carry the potent EXOmotifs, CGGGAG^[23]^ [Table 1].

### The HnRNP family in selective miRNA sorting into EVs: mechanisms, motif recognition, and biological relevance

Although the mechanisms underlying selective miRNA enrichment in exosomes remain incompletely understood, several members of the heterogeneous nuclear ribonucleoprotein (hnRNP) family have been identified as key regulators of post-transcriptional RNA metabolism and vesicle loading. These proteins are capable of binding specific RNA motifs, mediating transport, stabilization, and subcellular localization of miRNAs. For instance, hnRNPA2B1, hnRNPK, and hnRNPH1 have each been implicated in facilitating the sorting of miRNAs into exosomes through motif recognition or interaction with vesicle biogenesis pathways.

**Figure 3:**
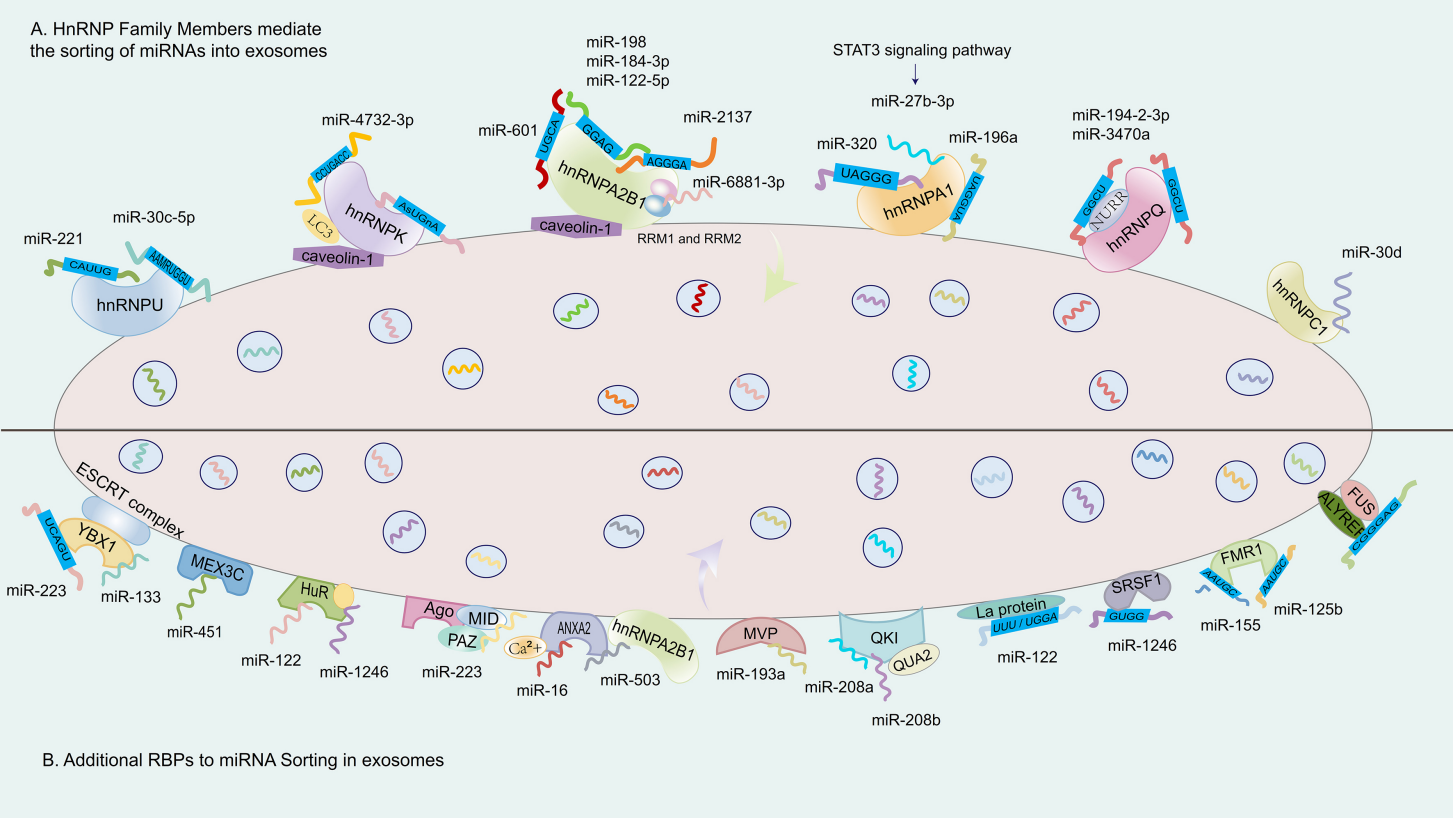
RBPs and miRNA sequences in the regulation of miRNA sorting into exosomes. (A) illustrates several representative RBPs from the hnRNP family, including hnRNPA2B1, hnRNPQ, hnRNPA1, and hnRNPU. These proteins bind to miRNAs by recognizing specific EXOmotifs (such as GGAG and CAUUG). This binding depends on the RRMs of the RBPs and may also be regulated by signaling pathways, such as the STAT3 signaling pathway; (B) shows that other RBPs, such as YBX1, HuR, and MVP, also form complexes with their target miRNAs by recognizing specific EXOmotifs. These complexes are directed to MVBs via intracellular transport mechanisms. Some RBPs, such as YBX1, can interact with the ESCRT machinery, thereby facilitating the selective loading of specific miRNAs into ILVs, which are ultimately released as exosomes. RBP: RNA-binding protein; hnRNP: heterogeneous nuclear ribonucleoprotein; hnRNPA2B1: heterogeneous nuclear ribonucleoprotein A2B1; hnRNPQ: heterogeneous nuclear ribonucleoprotein Q; hnRNPA1: heterogeneous nuclear ribonucleoprotein A1; hnRNPU: heterogeneous nuclear ribonucleoprotein U; EXOmotif: exosomal motif; STAT3: signal transducer and activator of transcription 3; YBX1: Y-box binding protein 1; HuR: human antigen R; MVP: major vault protein; MVB: multivesicular body; ESCRT: endosomal sorting complex required for transport; ILV: intraluminal vesicle; LC3: microtubule-associated protein 1 light chain 3; NURR: nuclear receptor receptor family.

Recent studies suggest that caveolin-1 can modulate hnRNPK activity through phosphorylation and O-GlcNAcylation, affecting its subcellular distribution and association with distinct EV subtypes^[83]^. HnRNPK has been reported to mediate the LC3-dependent sorting of miRNAs and was shown to interact with miR-4732-3p via a putative CCUGACC binding motif^[84]^ [Figure 3]. While these findings highlight a potential regulatory axis, caution is warranted: miR-4732-3p is not a canonical miRNA but rather a byproduct of the erythroid-specific miR-144/miR-451 cluster. It is highly abundant in red blood cells (RBCs) and virtually absent in other cell types under physiological conditions. Given that erythrocytes are prone to lysis during sample processing, the detection of miR-4732-3p in plasma-derived exosomes may result from contamination. Therefore, its purported enrichment in exosomes from non-erythroid cells, such as NSCLC lines, must be interpreted with care, as the observed effects could stem from technical artifacts rather than true biological function.

Among hnRNPs, hnRNPA2B1 has received the most extensive attention for its role in recognizing EXOmotifs, particularly the GGAG sequence, and facilitating miRNA inclusion into exosomes. It has been shown to interact with miR-122-5p in an m^6^A-dependent manner^[85]^ [Table 2], and to bind other miRNAs such as miR-198 and miR-601 via sumoylation-regulated recognition of 3’-motifs^[86,87]^ [Figure 3]. However, subsequent studies have extended hnRNPA2B1’s motif repertoire to include numerous inconsistent or poorly conserved sequences^[88]^, leading to questions about its selectivity and binding specificity. These inconsistencies are rarely reconciled across studies and are often presented as equally plausible models, despite a lack of rigorous comparative validation. Additional concerns arise from studies that report dramatic miRNA sorting disparities based on hnRNPA2B1 activity. For example, one report claimed that miR-2137 [Figure 3], a non-curated sequence not listed in miRGeneDB, accounted for over 20% of total EV miRNAs while comprising less than 0.5% of the intracellular pool^[89]^. Such disproportionate enrichment, in the absence of orthogonal validation, raises the possibility of non-physiological amplification or technical bias. Similar reports linking hnRNPA2B1 to miR-184-3p, miR-92a-2-5p, and miR-373-3p in multiple myeloma cells often lack mechanistic support and do not fully account for variables such as cell-type specificity, RNA degradation, or vesicle contamination^[67,90]^. Collectively, these examples illustrate the need for greater methodological stringency and critical evaluation in defining RBP–miRNA interactions. While hnRNPA2B1 remains a prominent candidate for miRNA sorting into exosomes, the wide variation in reported motifs, target miRNAs, and regulatory mechanisms underscores the complexity of this process. A more precise understanding will require the integration of orthogonal techniques such as CLIP-seq, single-EV RNA sequencing, and functional motif-disruption experiments. Only through such rigorous validation can the field distinguish genuine biological mechanisms from cell line– or technique-specific artifacts, ultimately guiding the rational design of EV-based RNA delivery systems.

**Table 2:** Various RBPs and the corresponding EXOmotifs involved in EV miRNA sorting

**RBPs**	**Regulated miRNAs**	**Recognized motif**	**Structural basis of RBP-motif interactions**	**References**
ALYREF	-	CGGGAG	Motif recognition	[23]
FUS				
hnRNPA2B1	miR-122-5p	GGAG	m6A modification-dependent	[85]
	miR-601		Motif recognition	[87]
	miR-198		Sumoylation modification-dependent	
hnRNPU	miR-221	CAUUG	Motif recognition	[93]
	miR-30c-5p	AAMRUGGU		[97]
hnRNPQ	miR-3470a	GGCU	NURR domain recognizes Motif	[100]
	miR-194-2-3p			[98]
hnRNPA1	miR-27b-3p	-	STAT3-dependent	[103]
	miR-196a	UAGGUA	Motif recognition	[104]
	miR-320	UAGGG (A/U)	Motif recognition	[105]
YBX1	miR-223	UCAGU	CSD domain recognizes Motif	[108]
	miR-133	-	H/R stress-induced	[121]
HuR	miR-122	-	Starvation stress-induced	[116]
Ago	-	-	PAZ/MID domains recognize 5’/3’ ends	[121]
	let-7a	-	KRAS/MEK/ERK pathway-dependent	[126]
QKI	-	-	QKI motif (ACU) -dependent	[135]
La protein	miR-122	UUU/UGGA	Motif recognition	[138]
SRSF1	miR-1246	CUGG	Motif recognition	[139]
FMR1	miR-125b	-	Hairpin structure recognition	[140]
	miR-155	AAUGC	Motif recognition	[141]

RBPs: RNA-binding proteins; EXOmotifs: exosomes enrichment; EV: extracellular vesicle; miRNAs: microRNAs; m6A: N6-methyladenosine; STAT3: signal transducer and activator of transcription 3; CSD: cold-shock domain; H/R: hypoxia/reoxygenation; Ago: argonaute; PAZ: piwi/argonaute/zwille; MID: middle; KRAS: Kirsten rat sarcoma viral oncogene homolog; MEK: MAPK/ERK kinase; ERK: extracellular signal-regulated kinase; QKI: quaking; SRSF1: serine/arginine-rich splicing factor 1; FMR1: fragile X mental retardation 1.

HnRNPC1 has been identified as a critical regulator of miR-30d enrichment in exosomes, with its expression level correlating with miR-30d loading efficiency [Figure 3]. Functional relevance has been demonstrated through co-culture experiments where hnRNPC1 knockdown, along with miR-30d knockout, led to impaired intercellular transfer of miR-30d during embryonic adhesion and invasion stages^[91,92]^. While this suggests a developmental role for hnRNPC1-mediated EV signaling, *in vivo* validation in model organisms is still lacking.

HnRNPU plays a broader role in EV-mediated miRNA transport, recognizing EXOmotifs such as CAUUG in miR-221 to promote its selective incorporation into exosomes^[93]^ [Figure 1 and Table 2]. The interaction between hnRNPU and miR-221 ensures efficient loading, emphasizing the important function of RBPs in regulating miRNA cargo within exosomes. This process is vital for intercellular communication and may offer significant therapeutic opportunities, as miR-221 is implicated in a variety of biological processes and disease pathways^[94-96]^. Apart from this, miR-30c-5p was the miRNA most strongly regulated by hnRNPU in terms of export. The sequence motif AAMRUGCU was notably enriched, indicating its role as a potential sorting signal^[97]^ [Table 2 and Figure 3]. However, these motif associations are primarily inferred from enrichment analyses, and the precise structural determinants underlying their recognition remain to be elucidated.

HnRNPQ, a liver-enriched RBP, has been associated with the loading of miR-3470a and miR-194-2-3p in hepatocyte-derived exosomes^[98,99]^ [Table 2]. Both miRNAs share the EXOmotif -GGCU, which is recognized by the NURR domain within hnRNPQ’s N-terminus^[99,100]^ [Figure 3]. Notably, miR-3470a is a non-canonical miRNA not annotated in major databases, raising concerns about potential misannotation or experimental artifacts. Another example involves hnRNPK, whose interaction with caveolin-1 facilitates its binding to miRNAs containing the AsUGnA motif^[101]^ [Figure 3]. Caveolin-1 appears to guide hnRNPK to membrane microdomains associated with EV formation. However, whether this interaction is required for motif-specific sorting remains speculative, as direct mechanistic data are limited. These studies highlight the diversity of RBP–miRNA sorting mechanisms, yet also reveal inconsistencies and technical challenges. Many studies rely heavily on correlative data, motif over-representation, or single-cell type systems. Moreover, several miRNAs reported as highly enriched (e.g., miR-3470a) are non-curated or tissue-restricted, raising the possibility of contamination or off-target effects. The relative physiological relevance of each RBP remains difficult to compare due to variations in experimental models, EV isolation protocols, and quantification methods.

HnRNPA1 has emerged as a multifaceted regulator of miRNA sorting into exosomes across various physiological and pathological contexts. Its regulatory influence appears to be mediated through both transcriptional signaling and direct motif recognition, although the specificity and consistency of these mechanisms remain under investigation. In the kidney, hnRNPA1 facilitates the packaging of miR-483-5p into urinary exosomes derived from renal tubular epithelial cells, helping regulate intracellular levels of this miRNA and thereby mitigating renal interstitial fibrosis progression^[102]^ [Table 1]. This represents one of the few examples where functional consequences of miRNA sorting into exosomes have been validated *in vivo*. Similarly, STAT3-activated hnRNPA1 promotes the export of miR-27b-3p into exosomes [Table 1], enhancing endothelial permeability, a process relevant to inflammation and vascular injury^[103]^ [Figure 3 and Table 2]. In both examples, upstream signaling modulates RBP activity, linking cell state to vesicle-mediated communication. Mechanistically, hnRNPA1 has also been shown to directly bind the UAGGUA motif at the 5’ end of miR-196a [Table 1], promoting its selective inclusion into exosomes, with downstream relevance in head and neck cancer models^[104]^ [Figure 3 and Table 2]. However, whether motif binding alone is sufficient to drive cargo selection, or requires cooperative cofactors or vesicular scaffolding proteins, remains unclear. The ability of hnRNPA1 to facilitate exosomal loading of miR-320 via UAGGG(A/U) recognition in chronic leukemia cells further supports its role in motif-guided sorting [Figure 3 and Table 2], though again, biochemical confirmation of direct binding and structural specificity is lacking^[105]^ [Table 1]. In tumor models, hnRNPA1 expression is often upregulated in cancer-associated fibroblasts (CAFs), where it correlates with enhanced loading of miR-522 into exosomes. This EV-mediated transfer of miR-522 suppresses ferroptosis in recipient cancer cells, contributing to a pro-tumorigenic niche^[106]^ [Table 1]. Likewise, under hypoxic conditions, glioblastoma cells exhibit increased hnRNPA1-dependent sorting of miR-1246 into cerebrospinal fluid-derived exosomes, a change associated with postoperative recurrence^[107]^ [Table 1]. While these data suggest stress-responsive hnRNPA1 activity, the mechanistic basis - whether sequence-specific or driven by global RBP redistribution - remains to be elucidated.

Despite the functional relevance of these miRNAs, several open questions remain. The diversity of reported binding motifs (e.g., UAGGUA, UAGGG, or motif-independent regulation via STAT3) raises concerns about binding specificity and methodological consistency across studies. In many cases, motif identification is based on enrichment analyses rather than direct structural validation, and EV quantification relies on bulk populations rather than single-vesicle resolution. Moreover, in tumor contexts, it is often difficult to dissociate true vesicular transfer from potential contamination or differential expression artifacts. Taken together, the current body of evidence highlights hnRNPA1 as a versatile RBP involved in the regulation of EV-mediated miRNA trafficking. However, the mechanistic heterogeneity, variable experimental models, and limited structural validation underscore the need for orthogonal approaches - such as CLIP-seq, structural mapping, and *in vivo* CRISPR knockout models - to define the rules governing hnRNPA1-dependent sorting and assess its therapeutic potential.

### The contributions of additional RBPs to miRNA sorting in exosomes

YBX1 (Y-box binding protein 1) is a well-characterized, multifunctional RBP whose cold-shock domain (CSD) specifically recognizes the 5’-proximal sequence UCAGU in miR-223 [Table 2], thereby promoting its selective sorting into exosomes derived from HEK293T cells^[108,109]^ [Figure 3]. Studies have revealed that phase separation–mediated cytoplasmic condensation of YBX1 and miR-223 facilitates their spatial colocalization, enabling efficient encapsulation into ILVs during membrane invagination within MVBs^[110]^. This model suggests a plausible mechanism for the selective and localized enrichment of RBP–miRNA complexes during EV biogenesis. Under stress conditions such as hypoxia/reoxygenation (H/R), YBX1 also contributes to the EV-mediated transfer of miR-133 from human endothelial progenitor cells [Table 2]. This miRNA enhances fibroblast angiogenesis and promotes mesenchymal–epithelial transition, thereby implicating YBX1 in stress-adaptive intercellular signaling^[111]^. Additionally, YBX1, in conjunction with the RNA methyltransferase NSUN2, has been shown to interact with specific RNA motifs - including ACCAGCCU, CAGUGAGC, and UAAUCCCA - in HEK293 S100 extracts. These sequence-specific interactions likely contribute to the recruitment of RNA cargo into exosomes, supporting a motif-guided mechanism of RNA sorting that may extend beyond miRNAs to other small non-coding RNAs^[112]^.

MEX3C (Mex-3 RNA binding family member C) is an RBP found in exosomes derived from both hepatocellular carcinoma (HCC) cells and normal hepatocytes^[113]^. It co-localizes with adaptor protein 2 (AP-2) and Argonaute 2 (AGO2), suggesting a potential role in the recognition or stabilization of specific miRNA–protein complexes during EV biogenesis. Functional studies have shown that silencing either MEX3C or AP-2 results in reduced levels of miR-451 in secreted exosomes, providing further evidence for MEX3C’s involvement in miRNA sorting^[114]^. However, the precise mechanism - whether through direct RNA binding, scaffold recruitment, or vesicle trafficking regulation - remains to be elucidated. In addition to its role in EV cargo selection, MEX3C has been implicated in clathrin-mediated endocytosis, whereas YBX1 interacts with components of the ESCRT machinery [Figure 3], suggesting that these two RBPs may participate in distinct but complementary pathways involved in vesicle formation and cargo loading. Importantly, phosphorylation-induced cytoplasmic accumulation of YBX1 has been shown to promote the secretion of pro-angiogenic factors and enhance the packaging of small non-coding RNAs into exosomes. This phosphorylation event may modulate its interaction with ESCRT-associated proteins, although the temporal dynamics and structural requirements of this process are still under investigation. These findings highlight how competitive or coordinated interactions between RBPs, such as YBX1 and MEX3C, may govern the selectivity and efficiency of RNA incorporation into exosomes. Further clarification of their respective roles - particularly regarding how they interface with vesicle budding and cargo recognition machinery - is essential for a deeper understanding of RNA sorting mechanisms.

HuR (Human antigen R) is a ubiquitously expressed RBP whose N-terminal region contains two RNA recognition motifs (RRMs) essential for binding to AU-rich elements in target RNAs^[115]^. This domain structure underlies HuR’s capacity to selectively interact with various non-coding RNAs under stress-related conditions. Notably, starvation-induced stress has been shown to promote the HuR-dependent accumulation of miR-122 in CD63-positive exosomes, with concomitant decreases in circulating miR-122 levels observed in mouse serum after 12 hours of starvation^[116]^ [Table 2]. In clinical samples, HuR also facilitates the sorting of miR-1246 into exosomes derived from the serum of gastric cancer patients^[117]^. The levels of miR-1246 are significantly elevated in exosomes from highly metastatic tumors relative to low-grade tumors, implicating HuR in metastatic potential and suggesting a stress-responsive role in EV cargo regulation^[117,118]^ [Table 1].

Ago2, a central effector in the RISC, is another key RBP involved in miRNA sorting. Its PAZ (Piwi/AGO/Zwille) and MID domains mediate precise recognition of the 5’ and 3’ ends of mature miRNAs [Table 1], enabling their incorporation into RISC and facilitating downstream gene silencing functions^[119-121]^ [Figure 3]. Ago2 plays an essential role in the miRNA sorting process^[122,123]^. Research indicates that Ago2 interacts with other RBPs and the ESCRT machinery, which is critical for the formation of MVBs and exosome biogenesis. Additionally, the secondary structure of miRNA duplexes plays a crucial role in the sorting of Ago proteins. Ago2 is widely recognized as the most prominent Ago protein^[124]^. Ago2 has been identified in multiple EV populations, including those released by platelets and colon cancer cells, where it forms stable complexes with miR-223 and other miRNAs^[125,126]^. In particular, the KRAS–MEK–ERK signaling axis negatively affects Ago2-mediated sorting of let-7a [Table 1], suggesting that post-translational signaling events can regulate the EV-targeting function of Ago2^[126]^. The structural properties of miRNA duplexes also influence Ago2 selectivity, although the full extent of this dependency is still under investigation. A notable finding is the dynamic interplay between HuR and Ago2 in miRNA regulation. These two RBPs may compete for overlapping miRNA targets, and their binding balance appears to be modulated by HuR ubiquitination, which is enhanced under stress conditions^[127]^. This suggests a broader regulatory network in which cellular stress not only alters RBP expression or localization but also rewires the miRNA packaging landscape via post-translational modifications and RBP–RBP interactions.

Annexin A2 (ANXA2) is a membrane-associated calcium-dependent protein that has been implicated in the selective sorting of miRNAs into exosomes. Recent studies have demonstrated that the abundance of miR-16 in exosomes is positively correlated with intracellular levels of ANXA2^[128]^. Overexpression of ANXA2 increases its presence in both cells and exosomes, leading to elevated levels of miR-16 in exosomes, while leaving intracellular miR-16 levels largely unchanged. This suggests that ANXA2 specifically regulates EV-mediated export of miR-16, rather than its transcription or stability [Figure 3]. Additionally, Ca^2+^ has been shown to modulate the binding efficiency between ANXA2 and miRNA, further highlighting a potential role for calcium signaling in tuning miRNA cargo loading. However, the precise structural mechanism through which ANXA2 recognizes miR-16 remains unclear and requires further investigation. Beyond direct binding, ANXA2 is also involved in cooperative miRNA export pathways. For example, it has been shown to interact with hnRNPA2B1, another RBP implicated in EV cargo sorting. This interaction appears to play a functional role in the export of miR-503 [Figure 3]. In endothelial cells, treatment with doxorubicin disrupts the hnRNPA2B1–miR-503 interaction, which paradoxically leads to an increase in EV-associated miR-503, possibly due to compensatory binding by ANXA2^[129]^. These findings suggest a context-dependent, competitive or cooperative relationship between ANXA2 and other RBPs in determining miRNA fate during EV biogenesis. However, further mechanistic clarification - particularly regarding RNA-binding specificity and vesicle localization - is necessary to validate these observations.

ALIX (ALG-2-interacting protein X) has been implicated in the selective loading of miRNAs into exosomes, particularly in exosomes secreted by human liver stem cell-like cells^[130]^. These ALIX-associated exosomes have been shown to be enriched in specific miRNAs, including miR-24, miR-31, miR-125b, miR-99b, and miR-221, which collectively exhibit anti-tumorigenic and regenerative properties *in vitro* and *in vivo* models^[86]^. While the exact mechanism remains incompletely understood, these findings suggest that ALIX facilitates the preferential incorporation of a defined subset of miRNAs, potentially through interaction with vesicle-forming complexes or RNA-protein scaffolds. This highlights ALIX as a noncanonical, but functionally relevant, contributor to EV cargo specificity.

Major vault protein (MVP), a component of one of the largest known cellular ribonucleoprotein complexes, has also been proposed to participate in EV-mediated miRNA sorting. Loss-of-function studies have demonstrated that MVP knockout leads to intracellular accumulation of miR-193a, reducing its secretion via exosomes and coinciding with suppressed tumor growth in certain cancer models^[131]^. These observations support the hypothesis that miRNA sorting into exosomes is not a passive process but rather an actively regulated event, likely involving non-canonical RBPs such as MVP. Furthermore, the tumor-suppressive effect observed from circulating exosomes underscores the biological and therapeutic relevance of precise miRNA cargo selection^[132]^. However, the molecular mechanism by which MVP mediates RNA recognition or vesicle targeting remains largely undefined.

QKI is an RBP involved in a wide range of post-transcriptional regulatory processes, including alternative splicing, RNA stability, and miRNA sorting. A key structural feature of QKI is the QUA2 domain, which enhances its ability to bind RNA by recognizing specific sequences such as the ACU motif [Table 2]. Loss of the QUA2 domain significantly impairs QKI’s RNA-binding capacity, underscoring its structural and functional importance^[72,133,134]^. In the context of cardiomyocyte biology, QKI has been shown to play a protective role against apoptosis, particularly under hypoxic or ischemic stress conditions. Recent studies have identified miR-208a and miR-208b as negative regulators of QKI: both miRNAs target the 3’ UTR of QKI mRNA [Table 1], leading to its downregulation^[135]^ [Figure 3]. Following H/R injury, these miRNAs are enriched in exosomes released by hypoxic cardiomyocytes, suggesting an EV-mediated transfer mechanism by which cardiomyocytes may propagate apoptotic signals to neighboring cells. The uptake of miR-208a/b-containing exosomes by recipient cardiomyocytes reduces QKI protein levels and disrupts its anti-apoptotic function, thereby exacerbating cardiomyocyte death under stress. This finding illustrates a feedback loop, where stress-induced miRNA expression suppresses a key protective RBP, with exosomes serving as the intercellular communication vector. However, the exact dynamics of miRNA sorting into exosomes and QKI’s broader role in vesicle biology remain to be fully elucidated. Further *in vivo* studies are necessary to confirm the physiological relevance of this pathway and assess its therapeutic potential in ischemic heart disease.

While TERT (telomerase reverse transcriptase) is categorized as a “non-classical RBPs”, its ability to bind specific RNAs is well-established, highlighting its role beyond telomere maintenance in regulating RNA-related processes^[136]^. Studies have shown that hnRNPF/H interacts with both TERT and its RNA components, influencing its activity and playing a role in cancer development as well as the proliferation and aging of human mesenchymal stem cells (MSCs)^[137]^. However, the precise mechanistic role of TERT in EV-mediated RNA sorting or secretion remains unclear. While its RNA interactions suggest a broader post-transcriptional regulatory capacity, direct evidence linking TERT to miRNA loading into exosomes is still lacking, and most current findings are derived from indirect observations or association studies.

The La protein, traditionally known for its role in RNA stabilization, has been shown to directly bind to UUU and UGGA motifs within miR-122 [Table 2], thereby promoting its loading into vesicles formed in cell-free systems^[138]^ [Figure 3]. This unusually high-affinity interaction suggests that La may act as a sequence-specific scaffold, although the physiological relevance of this finding remains to be confirmed *in vivo*.

Another RBP, SRSF1 (serine/arginine-rich splicing factor 1), has been identified as a regulator of miRNA cargo loading in pancreatic cancer cell-derived exosomes. RNA-sequencing analyses indicate that SRSF1 recognizes the CUGG motif and facilitates the enrichment of miR-1246 within exosomes^[139]^ [Table 2 and Figure 3]. While these findings offer novel insight into the intersection of spliceosome-associated factors and EV biology, it remains unclear whether SRSF1 directly binds mature miRNAs or exerts its effect via upstream RNA processing pathways. Further structural and functional validation is warranted.

In the neuronal context, FMR1 (fragile X mental retardation protein 1) is a well-studied RBP that preferentially recognizes hairpin structures. FMR1 has been reported to promote the EV-mediated export of miR-125b through its interaction with stem-loop RNA elements^[140]^ [Tables 1 and 2]. Moreover, FMR1 facilitates the loading of miR-155 by recognizing the EXOmotif AAUGC [Table 1], and is functionally linked to the ESCRT pathway in this context^[141]^ [Figure 3]. Interestingly, inflammasome activity has also been proposed to influence FMR1-mediated sorting of miR-155 [Table 2], although the exact signaling intermediates remain undefined. These findings collectively underscore the role of structure-specific and context-dependent RBP–miRNA interactions in EV biology, but also point to the need for standardized assays and functional models to dissect the temporal and spatial dynamics of these mechanisms.

In contrast, some miRNAs contain specific sequences known as CELLmotifs, which are associated with their retention within the cell. These motifs, often found in particular secondary structures or modified nucleotide sequences, promote the binding of miRNAs to RBPs. These RBPs play crucial roles in miRNA sorting and loading, but instead of facilitating packaging into exosomes, they help retain miRNAs inside the cell. The EXOmotif CAUGUG in sEV miRNAs from BAT cells and C[A/G][U/A]GG in AML12 cells both contain the shared core sequence CAUG^[23]^. In a similar way, the GGAG sequence is highly enriched in sEV/exosomal miRNAs derived from T cells. When the CELLmotif AGAAC was introduced into miR-431-5p, which exhibits low sEV enrichment, its presence in exosomes decreased by 35%^[23]^. Conversely, mutating the same AGAAC motif in miR-140-3p resulted in a twofold increase in its export to exosomes. Notably, disrupting the two core CELLmotifs (CAGU and AUUA) in miR-677-5p led to a remarkable 14-fold boost in its sEV enrichment^[23]^. These findings emphasize the pivotal role of specific motifs in directing miRNA sorting into exosomes.

In conclusion, the sorting of miRNAs into exosomes is a multifaceted and highly regulated process that involves a variety of molecular mechanisms. RBPs, miRNA motifs, the endosome-exosomes pathway, cellular states, and external stimuli all play essential roles in determining which miRNAs are packaged into exosomes. As our understanding of these mechanisms expands, it may become possible to engineer exosomes with increased specificity and efficiency for targeted therapeutic applications, improving the potential of exosomes-based therapies for a wide range of diseases.

## EXOSOMES CARGO LOADING FOR THERAPEUTIC APPLICATIONS

**Figure 4:**
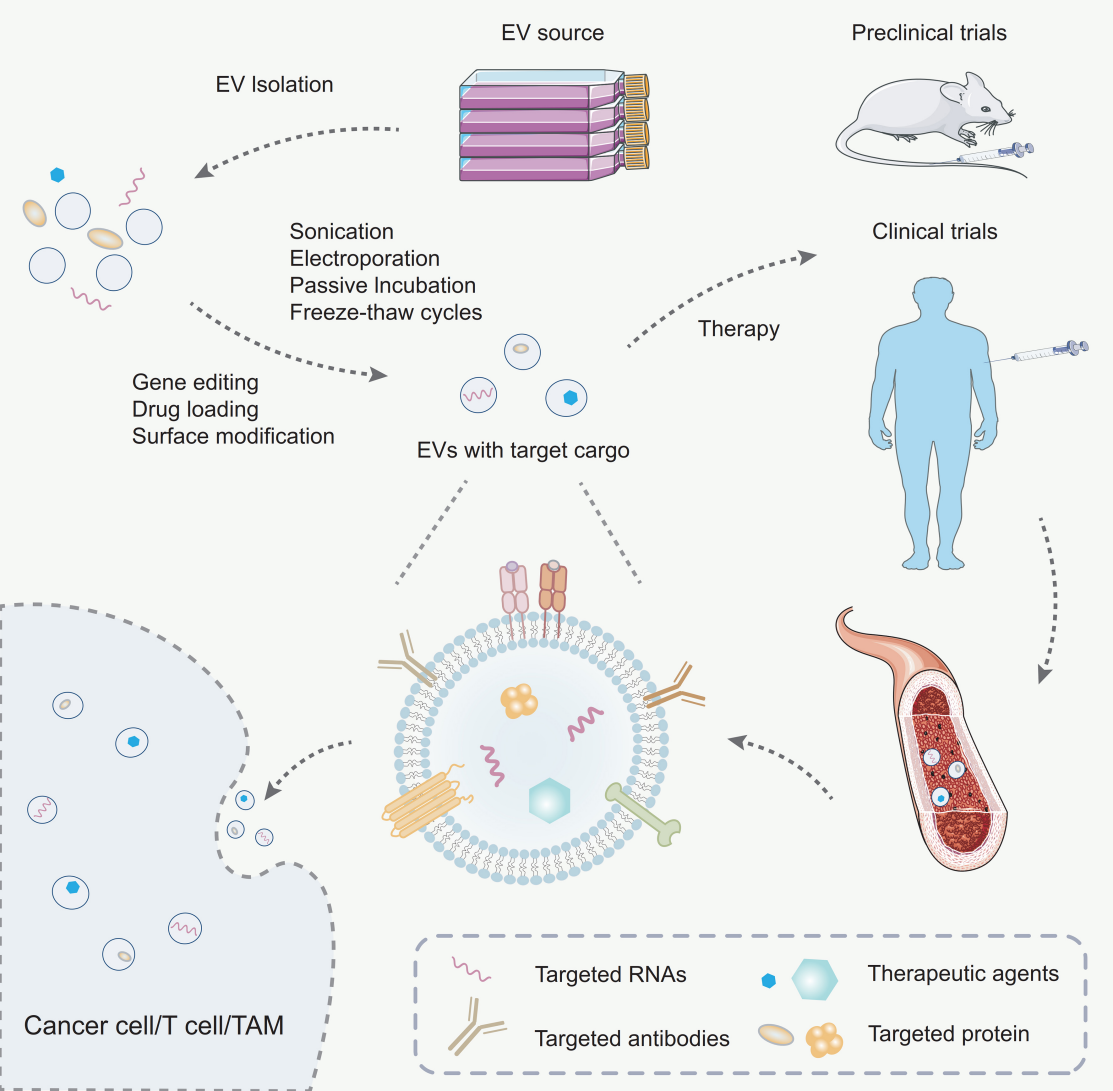
Engineering strategies for exosomes. Exosomes are first isolated from the culture supernatant via ultracentrifugation. To enhance their therapeutic potential, exosomes are subsequently loaded with specific cargo using either passive or active loading strategies. The engineered exosomes are then administered into murine or human vascular systems for therapeutic purposes. Once in circulation, these exosomes are capable of targeting specific cell populations such as T cells, tumor cells, or TAMs, thereby exerting targeted therapeutic effects. TAMs: Tumor-associated macrophages.

The initial objective of bioengineering exosomes was to incorporate small RNAs into these vesicles, inspired by the discovery that exosomes are capable of transporting RNA. However, due to an incomplete understanding of the mechanisms underlying RNA sorting into exosomes, early strategies primarily relied on cellular transfection - overexpressing target miRNAs within donor cells so that these molecules would be passively incorporated into exosomes and subsequently secreted for delivery^[142]^. As the field has evolved, a broad array of engineering approaches has emerged, generally categorized into four main types: biological (e.g., targeting peptides), immunological (e.g., antibodies), physical (e.g., magnetic particles), and chemical methods (e.g., sodium bicarbonate). Each of these techniques presents distinct advantages and limitations, prompting researchers to integrate multiple strategies to improve the targeting efficiency of engineered exosomes^[143]^. Among them, three foundational strategies - gene editing, drug loading, and surface modification - have proven especially critical in enhancing the therapeutic efficacy and targeting precision of EV-based delivery systems [Figure 4].

Gene editing is a cornerstone of EV engineering, enabling precise customization of their cargo by targeting the donor cells. Techniques such as CRISPR/Cas9 and RNA interference (RNAi) allow researchers to regulate specific genes, ensuring that therapeutic biomolecules like miRNAs, siRNAs, or proteins are effectively incorporated into exosomes^[144]^. Advanced approaches, including transcriptional and translational controls, further optimize cargo delivery to recipient cells. This precision enables exosomes to be tailored for specific therapeutic purposes, expanding their applications in gene therapy and regenerative medicine.

Loading therapeutic agents into exosomes is a pivotal step in their engineering. Exosomes can encapsulate a wide range of molecules, including small-molecule drugs, peptides, proteins, and nucleic acids such as siRNAs or miRNAs. Drug loading can be achieved through passive or active methods^[145]^. Passive loading involves simple incubation, allowing the drug to diffuse into exosomes naturally. In contrast, active loading employs advanced techniques such as electroporation, sonication, passive incubation, or freeze-thaw cycles to enhance drug encapsulation efficiency while maintaining EV integrity [Figure 4]. Furthermore, chemical conjugation methods allow drugs to be attached directly to EV surfaces, expanding the possibilities for targeted therapies. These drug-loaded exosomes have shown great potential in cancer treatment by delivering cytotoxic agents specifically to tumor cells, thereby minimizing systemic toxicity^[146]^.

Surface modification significantly enhances the functionality and stability of exosomes^[147]^. By engineering their surfaces, exosomes can be equipped with targeting ligands, peptides, antibodies, or aptamers to direct them to specific tissues or cell types. For example, surface modifications with tumor-targeting antibodies or integrin-binding peptides improve EV delivery to cancer cells, sparing healthy tissues. In addition, modifying the surface can enhance pharmacokinetics by increasing stability in circulation, reducing immunogenicity, or extending the half-life of exosomes in the bloodstream^[147]^. Techniques such as lipid conjugation, biotin-streptavidin systems, or genetic engineering of donor cells to express specific surface proteins have been effectively utilized. Moreover, adding stealth properties like PEGylation (polyethylene glycol) further shields exosomes from immune clearance, making them more efficient delivery systems^[148]^.

These strategies are often used in combination to create highly specialized exosomes tailored to specific therapeutic needs. By integrating these approaches, researchers have transformed exosomes into versatile tools for precision medicine, with applications ranging from targeted drug delivery and gene therapy to immunomodulation and diagnostics. For example, engineered exosomes derived from HCC cells and loaded with miR-26a have been shown to independently inhibit HCC progression both *in vitro* and *in vivo*^[149]^. These findings confirm the therapeutic potential of synthetic exosomal miR-26a and demonstrate the feasibility of using engineered exosomes as delivery vehicles^[149]^.

Importantly, emerging strategies now go beyond passive cargo loading. In particular, active loading of miRNAs through engineered RNA-binding motifs or sequence-specific recruitment domains has gained attention. A notable example is the study by Reshke *et al*., who engineered siRNA sequences into the Dicer-independent stem-loop structure of pre-miR-451, a microRNA naturally enriched in sEVs across various cell types^[150]^. This strategy enabled highly efficient EV-mediated delivery of siRNAs, resulting in robust gene silencing in the liver, intestine, and kidney of mice. Remarkably, the effective siRNA dose required was at least tenfold lower than that typically needed when using conventional lipid nanoparticle delivery systems. This approach highlights the potential of leveraging endogenous RNA motifs for enhanced and selective siRNA incorporation into exosomes, thereby improving therapeutic potency while minimizing dosage requirements^[150]^.

Furthermore, improving the *in vivo* stability and half-life of exosomes remains a key challenge. Surface PEGylation, low-immunogenic membrane modifications, and lipid-layer stabilization are among the leading strategies used to extend circulation time and reduce immune clearance, thereby enhancing the therapeutic window of engineered exosomes^[147,148]^. Recent studies have also highlighted the value of biomimetic membrane engineering in this context. For example, a comprehensive review has discussed the breakthrough applications of cell membrane- and EV-derived nanocarriers in treating osteoporosis (OP) and osteoarthritis (OA). By employing biomimetic strategies such as RBC membrane camouflage and targeted modification of exosomes, these nanoplatforms demonstrated markedly improved biocompatibility, prolonged systemic circulation (e.g., via PEG modification to evade immune clearance), and precise delivery to pathological sites using pH- or temperature-responsive release mechanisms. These findings underscore how membrane-derived EV platforms, when rationally engineered, can overcome many of the pharmacokinetic and targeting limitations currently facing EV-based therapeutics^[151]^.

EVs are emerging as versatile theranostic nanoplatforms due to their biocompatibility, low immunogenicity, and natural ability to carry bioactive cargo. As summarized by Xing *et al*., EVs can serve both as non-invasive biomarkers and as delivery vehicles for therapeutic molecules such as miRNAs, siRNAs, and proteins. The review also highlights innovative analytical tools - including microfluidic-based platforms and single-vesicle analysis technologies - that enable precise EV profiling and support clinical translation^[152]^. Furthermore, the authors discuss EV-based therapeutic strategies such as surface engineering, cargo enrichment, and cell reprogramming, while acknowledging key challenges like low loading efficiency, production scalability, and regulatory standardization. Despite these advantages, challenges such as loading efficiency, production scalability, and standardization for clinical translation remain key hurdles for future development.

While engineering efforts have significantly advanced the versatility of exosomes, clinical translation still faces critical hurdles. These include large-scale GMP-compliant production, standardized quality control, batch consistency, targeting efficiency *in vivo*, and unwanted immunogenicity. Addressing these challenges will require integration of scalable bioengineering techniques, biocompatibility assessment, and regulatory frameworks. Future efforts should also explore machine learning-guided EV design and liquid biopsy-integrated applications to optimize clinical readiness and disease-specific performance.

The bioengineering of exosomes involves the use of genetic techniques to create and customize exosomes with new functions and characteristics, grounded in the knowledge of their formation, release, and uptake processes. Currently, the strategies for exosomes encompass both genetic and non-genetic approaches for cargo loading and targeted delivery in vitro and *in vivo*. This review emphasizes EV bioengineering, focusing on molecular approaches to optimize cellular mechanisms that enhance EV production, sort cargo into exosomes within donor cells, and direct exosomes to target recipient cells. Exosomes have attracted significant interest for their role in transferring miRNAs and mRNAs between cells, facilitating intercellular communication. Their natural origin and unique biological characteristics of exosomes make them particularly well-suited as a specialized, efficient, and safe delivery system for therapeutic applications.

## CONCLUSION

While key details of EV biogenesis, release, and uptake remain unclear, progress in understanding cargo sorting has enabled efficient loading of therapeutic RNAs and proteins into engineered exosomes. Surface modification with ligands or receptors also enables targeted delivery to specific cells. The rapidly evolving field of EV engineering could further benefit from cross-disciplinary collaboration with artificial nanoparticle research, leveraging their similarities to advance therapeutic applications.

The elucidation of miRNA sorting mechanisms into exosomes not only deepens our understanding of EV biology but also provides critical insights into the selective nature of intercellular communication. Current evidence highlights that the loading of miRNAs into exosomes is a non-random, highly regulated process governed by RBPs (such as hnRNPA2B1, YBX1, SYNCRIP), specific sequence motifs (EXOmotifs), and post-transcriptional modifications like 3’ end uridylation and 2’-O-methylation. These mechanisms ensure the enrichment of certain miRNAs in exosomes, reflecting both the physiological state and the regulatory needs of the parent cell. Importantly, dysregulation of these sorting pathways has been implicated in pathological conditions, including cancer and neurodegenerative diseases^[153]^, where EV-derived miRNAs contribute to disease progression, immune modulation, and therapeutic resistance. Therefore, deciphering the rules governing miRNA packaging into exosomes not only facilitates biomarker discovery and disease diagnosis but also lays the groundwork for EV-based RNA therapeutics. In the broader landscape of EV research, miRNA sorting represents a frontier that bridges fundamental molecular biology with translational applications, emphasizing the need for more systematic, high-resolution approaches to fully map the determinants and dynamics of selective RNA cargo loading.

Key uncertainties persist regarding EV biogenesis, release, uptake, and cargo sorting. The roles of ESCRT-dependent and -independent pathways vary by cell type and state, requiring further study. The mechanisms balancing exosome secretion and lysosomal degradation, as well as the full scope of cargo sorting (especially for low-abundance miRNAs), remain unclear. Critically, the extremely low miRNA copy number per EV and undefined mechanisms for endosomal escape and functional activation raise questions about the biological significance of EV-contained miRNAs, suggesting their functional roles may be overestimated.

Furthermore, the multi-targeting nature of miRNAs further complicates delineating their precise regulatory functions. While some key players in MVB trafficking and EV release are known, many remain unidentified, and the selective exclusion of certain sorting proteins from exosomes highlights the need for deeper mechanistic insights. Exosomal surface molecules critically determine recipient cell recognition and uptake; however, the intracellular fate of exosomes and their cargo post-uptake is poorly understood. Addressing these fundamental questions necessitates innovative methodologies, particularly overcoming the current lack of high-resolution tools for accurately tracking cargo fate, quantifying individual EV content, and predicting single-vesicle functional outcomes. Integrating emerging technologies promises to clarify both the mechanisms of cargo release within recipient cells and the functional sufficiency of the delivered cargo to elicit biological responses.

To enable the clinical application of bioengineered exosomes, it is crucial to produce them using clinically approved cell types, such as human MSCs, embryonic stem cells (ESCs), and induced pluripotent stem cells (iPSCs). Scaling up EV production hinges on elucidating cell type-specific biogenesis and cargo sorting pathways. Existing bioengineering strategies, such as the EXOtic system developed for HEK-293T cells, often require optimization or adaptation for effective use in stem cells like hMSCs. Concurrently, enhancing targeting specificity beyond exosomes’ natural tropism for the liver and spleen is critical. Engineering EV surfaces with ligands or receptors offers a promising approach; refining this strategy through more precise targeting elements or incorporating multiple ligands per EV could significantly improve specificity and versatility for diverse therapeutic applications.

To improve reproducibility and translational relevance, EV research must adhere to standardized frameworks such as the MISEV2018 guidelines issued by the International Society for Extracellular Vesicles (ISEV), which define criteria for EV classification, isolation, and reporting^[154]^.

Looking ahead, future studies should focus on elucidating these underlying mechanisms, optimizing therapeutic cargo loading, and overcoming challenges in large-scale production and clinical application.

Altogether, exosomes - derived from cells - possess inherent biocompatibility, biological stability, and low immunogenicity, making them ideal candidates for therapeutic delivery. Advances in EV biology have laid the foundation for engineering strategies such as surface modification with targeting ligands and the manipulation of biogenesis pathways to enhance exosome yield and specificity. These developments collectively highlight the vast potential of EVs as next-generation delivery systems for diagnostics and RNA-based therapeutics.

## DECLARATIONS

### Copyright

© The Author(s) 2025.
